# A case of diverticulum of the appendiceal base resembling a submucosal tumor of the cecum under colonoscopy: a hitherto undescribed lesion

**DOI:** 10.1186/s12876-022-02337-3

**Published:** 2022-05-26

**Authors:** Toshihisa Kimura, Takanori Goi, Yuki Kidoguchi, Kenji Ohnishi, Tamotsu Togawa, Atsushi Iida, Yasunori Sato

**Affiliations:** 1 Department of Surgery, National Hospital Organization, Tsuruga Medical Center, 33-1, Sakuragaoka, Tsuruga, Fukui 914-0195 Japan; 2 First Department of Surgery, Faculty of Medicine, University of Fukui, 23-3, Matsuoka Shimoaizuki, Eiheiji-cho, Yoshida-gun, Fukui 910-1193 Japan; 3 Department of Human Pathology, Kanazawa University Graduate School of Medicine, 13-1, Takara-machi, Kanazawa, Ishikawa 920-8640 Japan

**Keywords:** Appendix, Diverticulum, Diverticulosis, Colonoscopy, Case report

## Abstract

**Background:**

Diverticulosis of the appendix is an uncommon clinical entity, and a preoperative diagnosis is often difficult. Herein we report an unusual case of appendiceal diverticulosis.

**Case presentation:**

A 72-year-old male was referred to our hospital to examine the cause of hematochezia. A colonoscopy study showed a protruding lesion resembling a submucosal tumor (SMT), approximately 20 mm in diameter, at the site around the appendiceal orifice of the cecum. An abdominal computed tomography and magnetic resonance imaging showed a cystic lesion at the appendiceal base. The lesion was clinically diagnosed as a cystic tumor of the appendix, but the possibility of a malignant tumor could not be excluded. Therefore, a laparoscopic ileocecal resection with lymph node dissection was performed. The pathological examination of the resected specimen revealed that the lesion was a diverticulum (pseudodiverticulum) occurring solitarily at the appendiceal base, in which the mucosal layer of the appendix was invaginated into the submucosa of the adjacent cecum, thus forming an SMT-like lesion.

**Conclusion:**

To our knowledge, this is the first case report in the English literature showing that an appendiceal diverticulum can manifest as an SMT-like lesion in the cecum. This condition should be recognized as a differential diagnosis for such lesions.

## Background

Diverticulosis of the appendix (DA) is an uncommon clinical entity, and it can present with symptoms similar to appendicitis. It is usually diagnosed after an appendectomy due to the difficulty in visualizing DA on imaging [1, 2]. While an appendectomy is an appropriate treatment for both DA and appendicitis, it is important to distinguish DA from appendicitis, as DA has an increased mortality risk due to perforation and relatively high rate in the coexistence of neoplasms [3, 4]. However, owing to its rarity and the lack of awareness related to its association with complicated appendicitis, DA remains underreported and poorly understood.

## Case presentation

A 72-year-old male was referred to our hospital because a fecal occult blood test was positive at the routine health check-up. His past medical history included a gastrectomy due to gastric ulcers at the age of 26 years, and a colonic polypectomy at the age of 66 years. His body height and weight were 163 cm and 67 kg, respectively. He presented with no symptoms of abdominal pain, vomiting or nausea. His abdomen was flat, soft and had no tenderness. His blood pressure, pulse rate and body temperature were 136/72 mmHg, 68 beats/min and 36.4 °C, respectively. Laboratory profiles including a complete blood count, and biochemical parameters, including the C-reactive protein level and tumor markers such as CEA and CA19-9 were all within normal limits.

**Fig. 1:**
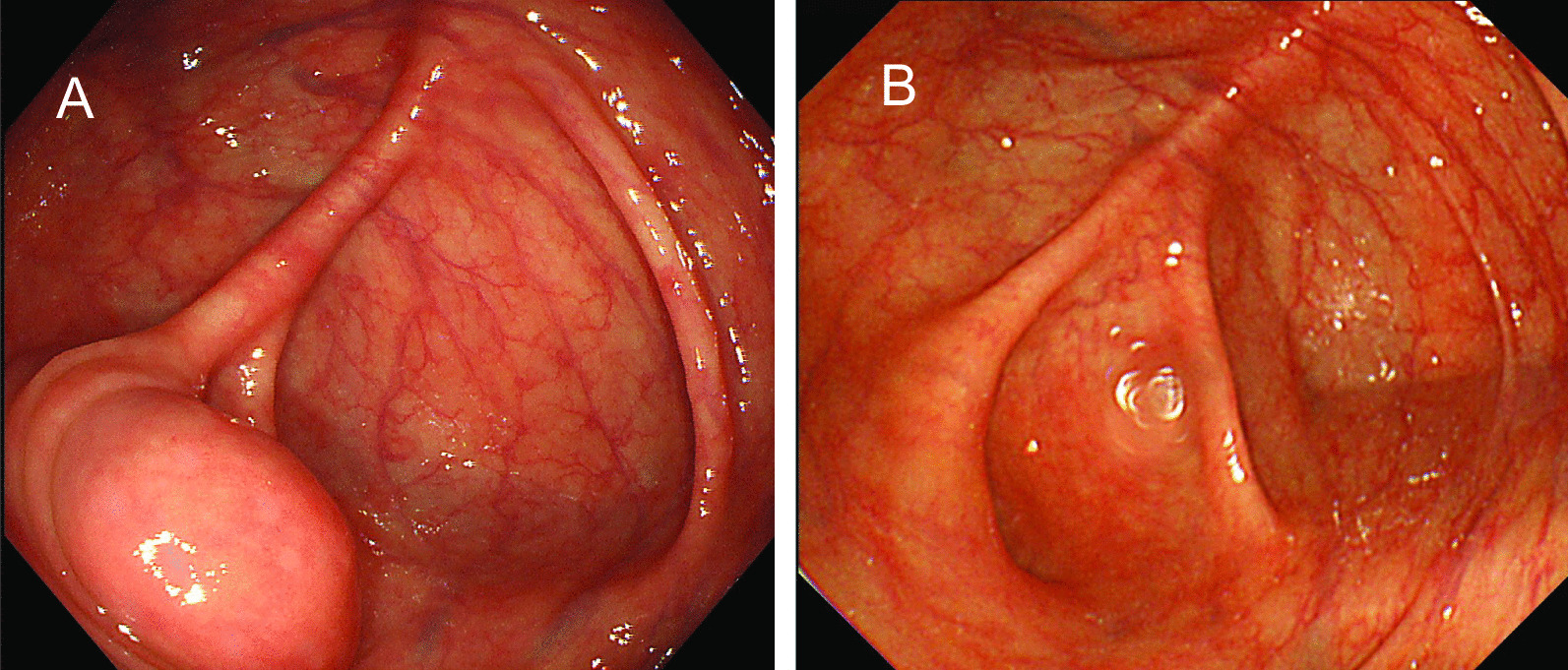
Colonoscopy findings. Colonoscopy performed just before surgery revealed a submucosal tumor-like lesion approximately 20 mm in diameter with a smooth surface at the cecum (**a**). Previous colonoscopy showing a normal cecum (5 years prior to surgery, **b**)

During the colonoscopy, a benign polyp was observed at the transverse colon, and it was endoscopically resected. And no diverticulum was observed on the entire colon. In addition, an SMT-like lesion, approximately 20 mm in diameter, with a smooth mucosal surface and a positive cushion sign was found in the cecum (Fig. 1a). The lesion was located at the site around the appendiceal orifice, although the appendiceal orifice itself could not be identified. The lesion had not been observed at the time of a previous colonoscopic examination performed 5 years prior (Fig. 1b).

**Fig. 2:**
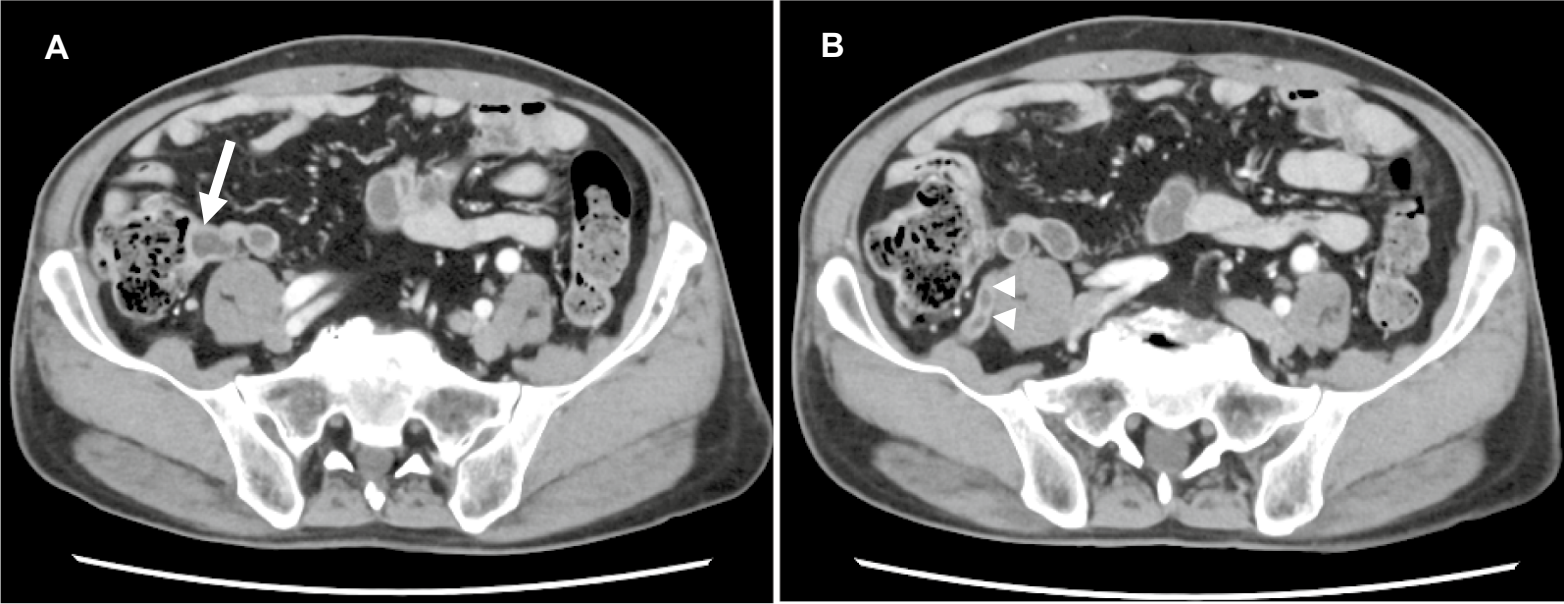
Abdominal computed tomography (CT) scan. Enhanced abdominal CT demonstrated a cecal cystic mass (**a**, arrow) without significant lymph node enlargement in the abdominal cavity. The appendix was almost normal size and do not have wall thickness or an appendicolith, but had a slightly dilation of the lumen (**b**, arrowheads)

**Fig. 3:**
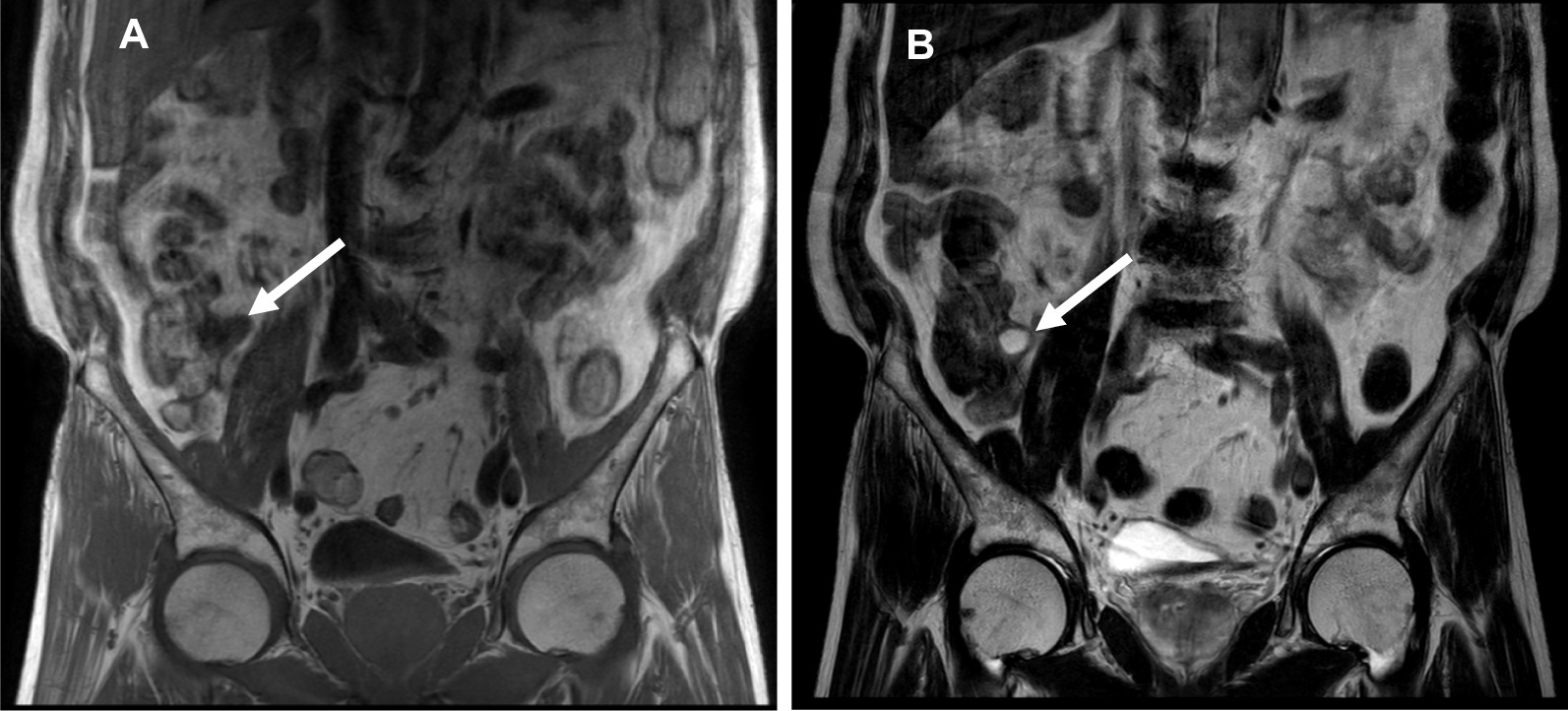
Abdominal magnetic resonance imaging (MRI) (coronal section). MRI revealed a cystic lesion at the appendiceal base with a low intensity on the T1-weighted imaging (**a**, arrow), and a high intensity on the T2-weighted imaging (**b**, arrow)

An enhanced abdominal computed tomography (CT) revealed a cystic lesion at the appendiceal base, and it occluded the cecal wall from the outside (Fig. 2a). The appendix was almost a normal size without any wall thickening or enhancement, but had a slightly dilation of the lumen (Fig. 2b). Enlargement of regional lymph node was not observed. Magnetic resonance imaging (MRI) revealed a cystic lesion at the appendiceal base with a low intensity on the T1-weighted images, and a high intensity on the T2-weighted images (Fig. 3). These findings indicated that the cystic lesion originated from the appendix, and the cystic lesions contained low viscosity fluid within it. The lesion was clinically diagnosed as an appendiceal cystic tumor, but the possibility of a malignant tumor could not be excluded. Therefore, a laparoscopic ileocecal resection after high ligation of the ileocolic artery with removed of nodes along the artery and its branches was performed after the consent of the patient. The patient had a favorable postoperative course and was discharged 7 days after surgery.

**Fig. 4:**
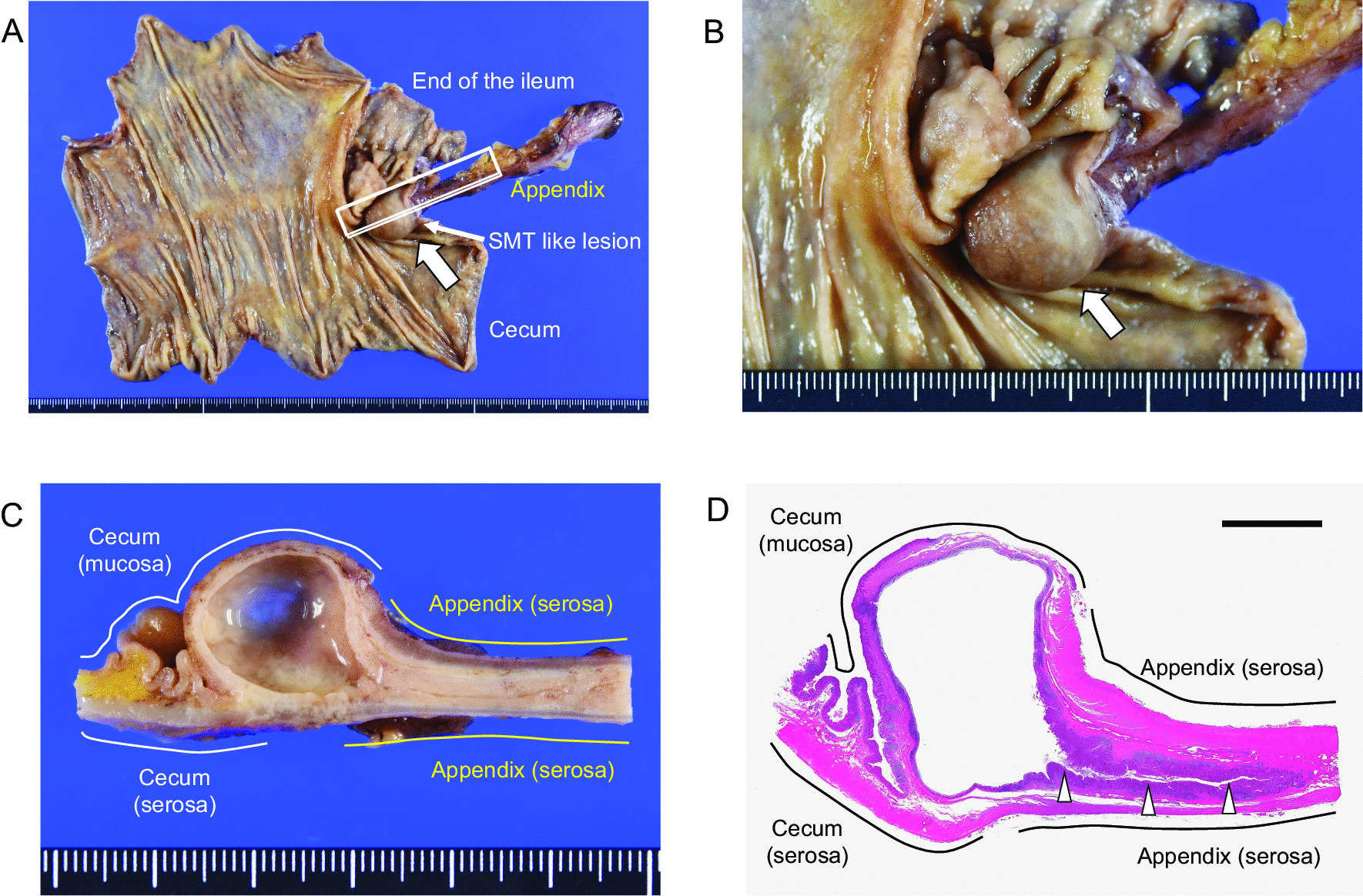
Macroscopic view of the resected specimen. **a**, **b** A submucosal tumor (SMT)-like lesion of the cecum (arrows). **c** Cut surface of the SMT-like lesion. The cut surface of the boxed area in **a** is shown in **c**. **d** Whole mount view of the SMT-like lesion (H&E staining). The lesion connected to the appendiceal lumen (arrowheads). The image of whole mount view was acquired by the use of a digital camera (EOS Kiss X10; Canon, Tokyo, Japan), followed by the processing using the Abode Photoshop software (Abobe, San Jose, CA). Scale bar; 1 cm (**d**)

The resected specimen grossly showed an SMT-like lesion, 20 × 20 mm in size, protruding out of the cecum at the site around the appendiceal orifice (Fig. 4a and b, arrows). The appendix was mildly enlarged over the entire length. The cut surface of the SMT-like lesion revealed a unilocular cyst filled with clear viscous fluid (Fig. 4c). The cyst was located at the appendiceal base, and there was a communication between the lumen of the cyst and the appendix (Fig. 4d), indicating that the lesion was formed by a cystic dilatation of the appendiceal lumen.

**Fig. 5:**
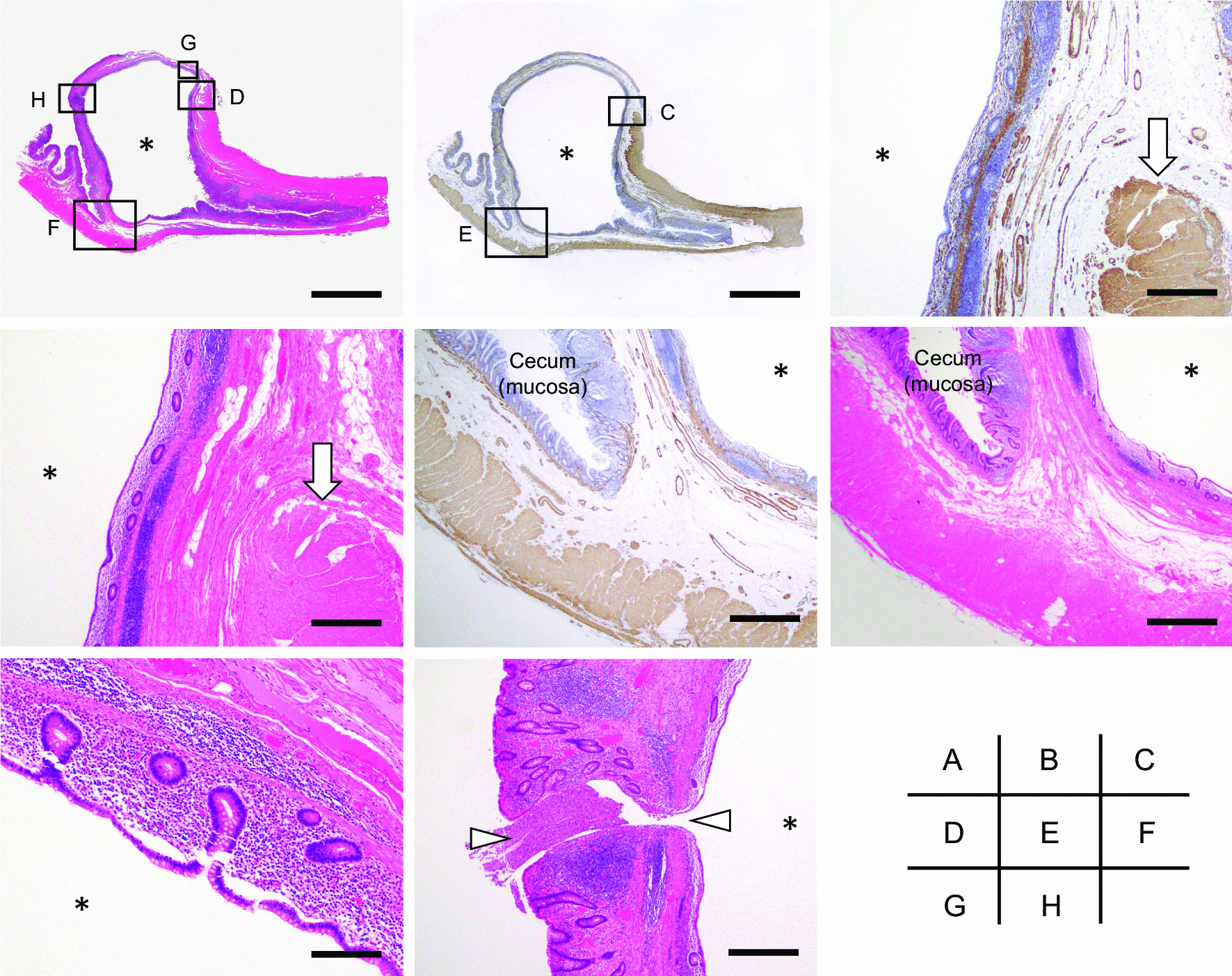
Histological findings of the lesion. **a** Whole mount view of the lesion (H&E staining). **b** Whole mount view of the lesion (immunostaining of alpha-smooth muscle actin). The close up images of the boxed areas with alphabetical indications in **a** and **b** are shown in each corresponding figure. **c**, **d** Disruption of the muscularis propria of the appendix (arrows). **e**, **f** Photographs showing the invagination of the mucosal layer of the appendix into the submucosa of the adjacent cecum. **g** The lumen of the cystic lesion was covered by nonneoplastic colonic mucosa. **h** Communication between the lumen of the cyst and the cecum (arrowheads). Asterisks indicate the lumen of the cystic lesion. Microscopic images were acquired by the use of a light microscopy (BX43; OLYMPUS, Tokyo, Japan) equipped with the image processing software cellSens (OLYMPUS). Scale bars; 1 cm (**a**, **b**); 2 mm (**c**, **d**, **h**), 1 mm (**e**, **f**); 0.5 mm (**g**)

Histologically, the muscularis propria of the appendix was disrupted at the origin of the cyst wall, which was more clearly observed in the sections of tissues that were immunostained for alpha-smooth muscle actin (Fig. 5a–d, arrows). At the site of the disruption, the mucosal layer, including the lamina propria and the muscularis mucosa, invaginated into the submucosa of the adjacent cecum (Fig. 5c–f). The lumen of the lesion was entirely covered by the normal mucosa (Fig. 5g). Epithelial proliferation and hypermucinous epithelium, which were consistent with a low-grade appendiceal mucinous neoplasm (LAMN) were not observed. Based on these findings, a pathological diagnosis of a diverticulum (pseudodiverticulum) of the appendiceal base was made. The histological examination showed no signs of inflammation indicative of diverticulitis and there were no other diverticula in the appendix. Although the site of the appendiceal orifice was grossly unclear, it was identifiable under a light microscope (Fig. 5h, arrowheads). Histological findings indicative of malignancy were not observed in a total of 20 dissected lymph nodes.

## Discussion and conclusions

DA is an uncommon clinical entity, and the incidence of DA found in appendectomy specimens ranges from 0.004 to 2.1% [5, 6]. Diverticula are classified into congenital and acquired diverticula. Congenital diverticula are true diverticula resulting from an abnormal bowel recanalization during the solid phase [7, 8], and are associated with “D” trisomy or cystic fibrosis [9, 10]. Congenital diverticula are very rare, and are characterized by an invagination of the entire intestinal wall including the mucosa, submucosa and muscularis propria into a normal intestinal wall [11]. In contrast, acquired diverticula are caused by invagination of the mucosal and submucosal layers through weakened portion of the intestinal wall, and they occur due to an increased intraluminal pressure [3]. Because acquired diverticula pathologically lack the muscularis propria, they can be easily perforated due to the delicacy of the tissue.

DA has been classified into 4 distinct subtypes by Phillips et al. [12]. Type 1 occurs in a normal appendix associated with acute diverticulitis. Type 2 is characterized by diverticulitis with an underlying acute appendicitis [13]. Type 3 represents acute appendicitis with incidental diverticula. Type 4 is defined as diverticula without either appendicitis or diverticulitis, as was seen in the present case. Among the four types, Type 4 is the most difficult lesion to identify because it is the only type that is asymptomatic. In general, DA is diagnosed after an appendectomy because the diverticulum is usually small and located in the mesenteric border, and diverticulitis presents with clinical features that are similar to acute appendicitis.

The challenge in diagnosis of DA preoperatively is mainly due to the difficulty in visualizing the DA on imaging [1, 2]. There are several reports that describe the radiological findings in DA, and CT is reported to be the most useful modality for the diagnosis of DA [14, 15]. CT can detect appendiceal diverticulitis based on findings such as an increased density of pericecal fat, the absence of fluid collection in the appendix, the absence of an appendicolith, and the formation of abscesses [14, 15]. In the present case, however, these findings were not observed due to the patient not having diverticulitis.

Although an acquired diverticulum is usually small in size (2–5 mm), this case exhibited a large cystic lesion (the maximal diameter was 20 mm) [16]. It has been reported that large appendiceal cysts are more likely to be neoplastic, and LAMN is the most likely diagnosis in these cases, even though the present case did not have a LAMN [17]. The reason that this case formed large cystic lesion without rupturing maybe as follows: a small appendiceal diverticulum first developed at the site around the appendiceal orifice. It blocked the flow of discharge from the appendix, leading to an increase in the appendiceal intraluminal pressure, and the sustained high pressure made the lesion larger. Moreover, due to the presence of the rare case where the appendiceal diverticulum was invaginated into the submucosa of the adjacent cecum, the surface of diverticulum was covered by the cecal mucosa, and this covering of the cecal mucosa prevented the lesion from rupturing. Generally, asymptomatic DA is not an indication for surgery, but a prophylactic appendectomy may be beneficial to prevent complications related to DA. On the other hand, DA is reported to be occasionally accompanied by neoplasms, especially LAMN and carcinoid tumors [3, 4]. Additionally, several cases of pseudomyxoma peritonei associated with DA have been reported [18].

As for this case, although there were few findings to doubt malignancy*,* we could not exclude the possibility of malignant tumor, especially mucinous neoplasm. Appendiceal mucinous neoplasms include mucinous adenocarcinoma (MAC) and LAMN according to the World Health Organization classification of tumours [19]. We doubted the possibility of LAMN rather than MAC, because the CT scan revealed neither irregular wall thickening nor nodules in the cystic lesion. The operation procedure for appendiceal mucinous neoplasm is controversy and the treatment strategy has not been established. However, as long as the possibility of mucinous neoplasm could not be ruled out, neither simple appendectomy nor simple cecectomy could be a surgical option. Otherwise, when we choose colon resection with lymph node dissection, dissection area is not defined for the treatment of appendiceal mucinous neoplasm [20]. In other words, which one to select is controversial an ileocecal resection with lymph node dissection or a right hemicolectomy with lymph node dissection. After the consent of the patients we selected an ileocecal resection with lymph node dissection for the surgical procedure rather than a right hemicolectomy with lymph node dissection for the purpose of performing just enough surgery, because enlargement of regional lymph nodes was not observed in the preoperative assessment, and lymph node metastasis is rare in LAMN [21].

A rare case of DA with unusual endoscopic and pathologic futures was reported. Based on its characteristic appearance during colonoscopy, the lesion was first suspected to be SMT of the cecum. To our knowledge, this is the first case report in the English literature showing that DA can manifest as an SMT-like lesion in the cecum. This condition should be recognized as a differential diagnosis for such lesions.

## Data Availability

Not applicable.

## Abbreviations

CT: Computed tomography
DA: Diverticulosis of the appendix
LAMN: Low-grade appendiceal mucinous neoplasm
MAC: Mucinous adenocarcinoma
MRI: Magnetic resonance imaging
SMT: Submucosal tumor
